# Morphological and Physiological Plasticity of Spinal Lamina II GABA Neurons Is Induced by Sciatic Nerve Chronic Constriction Injury in Mice

**DOI:** 10.3389/fncel.2018.00143

**Published:** 2018-05-24

**Authors:** Hongmei Zhang, Yan Li, Qing Yang, Xian-Guo Liu, Patrick M. Dougherty

**Affiliations:** ^1^Division of Anesthesiology and Critical Care Medicine, Department of Pain Medicine, University of Texas MD Anderson Cancer Center, Houston, TX, United States; ^2^Department of Integrative Biology and Pharmacology, The University of Texas Health Science Center at Houston, Houston, TX, United States; ^3^Department of Physiology and Pain Research Center, Zhongshan School of Medicine, Sun Yat-sen University, Guangzhou, China

**Keywords:** neuropathic pain, disinhibition, patch clamp, IPSC

## Abstract

Mice with transgenic insertion of code for enhanced green fluorescent protein (EGFP) at the locus for glutamic acid decarboxylase 67 (GAD67), one of two key enzymes for the synthesis of γ-aminobutyric acid (GABA) were used to test whether the morphological properties of these neurons show plasticity with nerve injury. Physiological properties and the delivery of intracellular label to EGFP-expressing lamina II neurons was done using whole-cell patch-clamp in spinal cord slices from sham and chronic constriction injury (CCI) mice. As well, whole cell recordings were made of non-EGFP labeled cells to ascertain changes in overall inhibitory signaling following CCI. The EGFP labeled neurons in both sham and CCI mice exhibited islet, central and vertical cell morphological profiles but no radial cell profiles were observed. The length of cell dendrites was found to be significantly shorter in CCI mice for all cell profile types. The longest neurites averaged 155.96 ± 18.29 μm in CCI mice compared to 334.93 ± 29.48 μm in sham control mice. No change was observed in either passive or evoked membrane properties of EGFP-expressing neurons in CCI versus sham mice. Meanwhile, the frequency of miniature inhibitory post-synaptic currents of non-EGFP expressing spinal lamina II neurons was significantly reduced. These results suggest that reduced inhibitory output from GABA neurons occurs with nerve injury in part due to altered cell morphology.

## Introduction

Spinal Lamina II has long been known to play a role in modulating incoming sensory afferent information (Light and Perl, 1979; Sugiura et al., 1986); and this process is becoming better defined as specific subpopulations of excitatory and inhibitory neurons and their connectivity is becoming revealed (Peirs et al., 2015; Todd, 2017). Anatomical studies have shown that roughly one third of lamina II neurons contain the inhibitory neurotransmitter γ-aminobutyric acid (GABA) (Todd and Sullivan, 1990; Polgar et al., 2003). GABA is synthesized from glutamate by the enzyme glutamic acid decarboxylase (GAD) and so can be used as a marker for these inhibitory interneurons. GAD has two isoforms, GAD67 and GAD65, each encoded by a different gene, gad1 and gad2, respectively (Erlander et al., 1991). GABA neurons form synapses on primary afferent Aδ-fiber and C-fiber terminals, mediating presynaptic inhibition (Todd, 2010); and GABA is also mediates post-synaptic inhibition to spinal dorsal horn neurons (Magoul et al., 1987; Bernardi et al., 1995). Spinal application of the GABA_A_ receptor antagonist bicuculline produces behavioral signs touch evoked pain (Yaksh, 1989; Malan et al., 2002) and enhanced reflex activity (Sivilotti and Woolf, 1994); while GABA receptor agonists decrease pain-like behavior after nerve injury (Malan et al., 2002).

There are multiple alterations in the dorsal horn subsequent to nerve injury that play key roles in driving and maintaining the accompanying neuropathic pain. Among these, dysfunction in inhibitory signaling in spinal dorsal horn has several lines of evidence that this is an important contributor to neuropathic pain (Todd, 2015). GABA immunoreactivity is decreased (Ibuki et al., 1997; Eaton et al., 1998) and both afferent evoked and spontaneous inhibitory post-synaptic potentials are reduced in the spinal dorsal horn after nerve injury (Moore et al., 2002). It has been suggested that reduction in spinal inhibition is due to outright loss of inhibitory neurons (Castro-Lopes et al., 1993; Ibuki et al., 1997; Moore et al., 2002; Scholz et al., 2005; Meisner et al., 2010); while others have suggested that the only apoptosis of cells in the spinal cord is due to loss of microglia (Polgar et al., 2003, 2004, 2005; Polgar and Todd, 2008). The properties of identified spinal GABA neurons following nerve injury has been facilitated by the generation of mice expressing code for enhanced green fluorescent protein (EGFP) at the GAD67 locus. In the context of multiple measures of reduced inhibitory processes in the spinal cord with nerve injury, it was surprising that the excitability and discharge properties of GABA neurons were unchanged in nerve injured mice (Balasubramanyan et al., 2006; Schoffnegger et al., 2006). An alternate possibility that might explain decreased spinal inhibitory processes following nerve injury is that GABA neurons alter their morphology that results in altered processing of inputs and outputs with neighboring neurons. In this study, GAD67-EGFP transgenic mice were used to test whether the morphological properties of spinal GABA neurons show plasticity with nerve injury.

## Materials and Methods

### Animals

A breeding pair of transgenic EGFP-GAD67 mice on a C57BL/6 background (Heinke et al., 2004) were purchased from Jackson laboratories [FVB-Tg(GadGFP)45704Swn/J, Stock # 00317, Bar Harbor, ME, United States] and bred in the animal facility of The University of Texas MD Anderson Cancer Center. The transgene expression was confirmed in homozygous breeding pairs using RT-PCR and in offspring by microscopic visualization of fluorescent neurons. All the surgical and experimental protocols were approved by the Animal Care and Use Committee of MD Anderson Cancer Center and conformed to the NIH guidelines on the ethical use of animals. The animals were housed in environment controlled conditions on a 12:12 light–dark cycle in standard cages with approved bedding and with food and water available *ad libitum.* All efforts were made to minimize the number of animals used and their suffering.

### CCI Model and Behavior Test

Mice of both sexes between 6 and 7 weeks of age (25–30 g) were deeply anesthetized with 2–3% isoflurane. The left sciatic nerve was exposed under aseptic conditions at the mid-thigh level proximal to the trifurcation and freed from adhering tissue. Three ligatures (chromic gut, 6-0) were tied around the nerve with about 1 mm spacing in between similar to procedures previously used on rats (Carlton et al., 1991; Garrison et al., 1991; Dougherty et al., 1992; Palecek et al., 1992) as done previously in the lab (Zhang et al., 2013). The muscle and skin incisions were then closed. In sham animals, the left sciatic nerve was exposed and freed from the connective tissue as in the chronic constriction injury (CCI) mice but not surrounded with suture. The responses of mice to mechanical stimulation of both hindpaws (ipsilateral and contralateral to CCI) were evaluated daily beginning 3 days before CCI and then at days 1, 3, and 7 after surgery. Animals were placed under acrylic boxes that were atop wire mesh floors and allowed to habituate for 1 h. Von Frey filaments were applied to the plantar surface of paw and the withdrawal threshold measured using an up–down method beginning with a 0.6 g filament (Chaplan et al., 1994). CCI mice with confirmed mechanical hypersensitivity in the ipsilateral paw (compared to the contralateral paw) and sham mice exited the behavioral studies to terminal electrophysiological and morphological experiments at day 3 to day 7 after surgery.

### Spinal Cord Slice Preparation

The mice were anesthetized with 2% isoflurane, and the lumbar segment of the spinal cord was rapidly removed following partial laminectomy. The mice were then euthanized by inhalation of 5% isoflurane followed by cervical dislocation and exsanguination. The spinal cord segment was immediately placed in an ice-cold sucrose artificial cerebrospinal fluid (ACSF) solution pre-saturated with 95% O_2_ and 5% CO_2_ that contained: 234 mM sucrose, 3.6 mM KCl, 1.2 mM MgCl_2_, 2.5 mM CaCl_2_, 1.2 mM NaH_2_PO_4_, 12.0 mM glucose, and 25.0 mM NaHCO_3_. The tissue was then placed in a shallow groove formed in a gelatin block and glued onto the stage of a Vibratome (Leica, VT1200, Buffalo Grove, IL, United States). Transverse or parasagittal spinal cord slices (300 μm) were cut in the ice-cold sucrose ACSF and pre-incubated in Krebs’ solution oxygenated with 95% O_2_ and 5% CO_2_ at 34°C for at least 1 h before they were transferred to the recording chamber. The Krebs’ solution contained 117.0 mM NaCl, 3.6 mM KCl, 1.2 mM MgCl_2_, 1.5 mM CaCl_2_, 1.2 mM NaH_2_PO_4_, 11.0 mM glucose, and 25.0 mM NaHCO_3_. Each slice was placed in a glass-bottomed chamber (Warner Instruments, Hamden, CT, United States) and fixed with parallel nylon threads supported by a U-shaped stainless steel weight. The slice was continuously perfused with Krebs’ solution at 3.0 ml/min at 34°C maintained by an inline solution heater and a temperature controller (TC-344B; Warner Instruments, Hamden, CT, United States).

### Electrophysiological Recordings

Neurons located in lamina II were identified under a fixed stage microscope (BX51WI; Olympus, Tokyo, Japan) with differential interference contrast/infrared and epifluorescence illumination. The electrode for the whole-cell recordings was triple pulled from borosilicate glass capillaries with a horizontal puller (P-97; Sutter Instrument Company, Novato, CA, United States). The impedance of the pipette was 8 to 12 MΩ when filled with internal solution containing: 135.0 mM K-Gluconate, 5.0 mM KCl, 2.0 mM MgCl_2_, 0.5 mM CaCl_2_, 5.0 mM HEPES, 5.0 mM EGTA, 5.0 mM ATP-Mg, 0.5 mM Na-GTP, adjusted to pH 7.2 to 7.4 with 1 M KOH (290–320 mOsm). The input resistance was monitored, and the recording was abandoned if this changed by more than 15%. Signals were amplified using a MultiClamp700B amplifier (Axon Instruments, Foster City, CA, United States) filtered at 2 kHz, and digitized at 10 kHz using a Digidata 1440 interfaced with a personal computer. Access resistance, typically 15–30 MΩ, was measured after whole-cell configuration was established and monitored throughout the recording. The cells were abandoned if the access resistance changed more than 20%. Current clamp recordings of EGFP+ neurons were made in bridge mode with liquid junction potential corrected. Only cells with resting membrane potential (RMP) more negative than -45 mV were collected. Two minutes of spontaneous activity was collected at the beginning of each recording. Neurons were classified as whether they showed spontaneous action potential discharges (spontaneous firing) or were silent. The firing patterns of neurons were determined by their responses to a series of depolarizing currents injected to the recorded neurons through patch pipette (from 0 to 400 pA with 20 pA increment, 1 s duration). The current threshold was defined as the minimal current that evoked an action potential. In a separate set of experiments, mIPSC’s were measured in non-EGFP expressing neurons in voltage clamp mode at a holding potential of 0 mV and with the addition of 0.5 μM tetrodotoxin added to the bath solution.

### Intracellular Labeling

In a subset of experiments, parasagittal spinal cord slices (300 μm) were used and neurobiotin (0.5%, Vector Laboratories, Burlingame, CA, United States) was added in the patch pipette solution and allowed to diffuse into the target neurons for 20–30 min. The patch pipette was carefully withdrawn from the recorded neuron and the slice was stored in 4% buffered paraformaldehyde overnight. Slices were rinsed 3x 10 min with phosphate buffered saline (PBS) and treated with blocking solution (PBS containing 0.2% Triton X-100, 2% BSA, and 3% NGS) for 1 h. Slices were then incubated with Cy3 steptavidin (1:200) in blocking solution for 4 h at room temperature in the dark. Slices were then rinsed 3x 10 min with PBS and mounted on a glycerin-based medium slides. Three-dimensional reconstructions of the filled neurons were made using the FV1000 confocal microscopy (Olympus BX-UCB, Tokyo, Japan). All images were taken using identical acquisition parameters and final representative figures are presented as the original images without further modification. Neuronal profiles were traced using Image J (National Institutes of Health). The longest neuronal branch was measured from the edge of soma to the most distal tip. In some cases several measurements were made to determine which process was longest, but only the longest one was used for analysis. The number of branches on the longest identified processes were also measured as was the longest unbranched segment.

### Data Analysis

Group sizes were based on power analysis using parameters from previous neurophysiological studies (Zhang et al., 2013). Data were expressed as mean ± standard error of mean (SEM) and analyzed with GraphPad Prism 5. Differences between means were tested for significance using non-paired *t*-test. *p* < 0.05 was considered statistically significant.

## Results

### CCI Induced Mechanical Hypersensitivity in Mice

**FIGURE 1:**
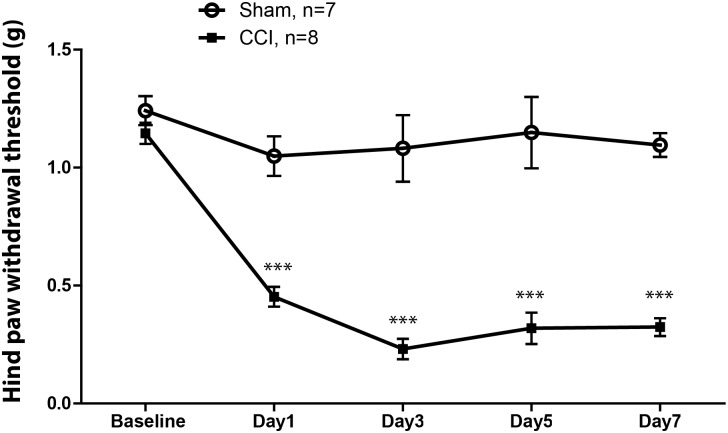
Mechanical withdrawal threshold of the hindpaw in mice ipsilateral to chronic constriction injury (CCI) of sciatic nerve (filled squares) and that of the hindpaw in mice ipsilateral to sham injury (open circles). The withdrawal threshold in mice with the CCI was significantly reduced at post-operative day 1, and remained significantly lower across the time interval observed. The threshold of ipsilateral hindpaw withdrawal showed no changed in sham mice. ^∗∗∗^*p* < 0.001.

The withdrawal threshold to mechanical stimulation showed a significant decrease in the paw ipsilateral to the CCI compared with the contralateral paw (**Figure 1**) and to withdrawal threshold in sham mice (not shown) by 1 day after surgery; and this difference lasted at least 7 days similar to that previously observed in the laboratory (Zhang et al., 2013).

### Physiological Properties of EGFP-GAD67+ Neurons in CCI Mice

Spinal cord slices from L4 to L6 were acutely prepared and used to investigate membrane and discharge properties of GABA neurons identified in spinal lamina II by visualization of green fluorescence. Whole cell patch-clamp recordings were obtained in a total of 104 GABA neurons from lamina II of spinal cord; 56 from 16 CCI mice and 58 from 15 sham mice. The RMP, membrane resistance, cell capacitance, and spike-burst responses to intracellular current injection were measured 2 min after establishment of whole cell configuration. As summarized in **Table 1** there were no differences in these properties between CCI and sham mice.

**Table 1:** Passive and active membrane properties of spinal GABA neurons in mice with a sham nerve injury (Sham) or sciatic nerve chronic constriction injury (CCI).

		Sham (*n* = 29)	CCI (*n* = 28)
Membrane potential (-mV)		60.9 ± 1.6	60.0 ± 1.8
Membrane resistance (MΩ)		463.7 ± 25.2	449.1 ± 26.3
Cell capacitance (pF)		38.8 ± 1.1	38.1 ± 1.2
Rheobase (nA)		282.7 ± 26.3	278.8 ± 28.8
Action potential pattern	Spontaneous	37.9% (11)	25.0% (7)
	Tonic	10.3% (3)	10.7% (3)
	Initial	20.7% (6)	7.1% (2)
	Delayed	10.3% (3)	21.4% (6)
	Single	6.9% (2)	14.3% (4)
	Irregular	13.8% (4)	21.4% (6)

*The numbers in parenthesis are the actual number of cells observed.*

**FIGURE 2:**
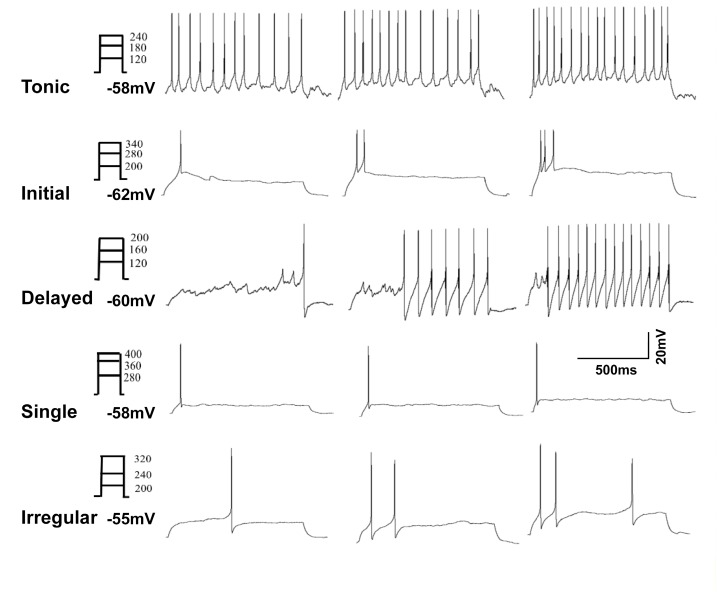
Representative analog recordings show the five types of firing patterns evoked by injection of depolarizing current (0–400 pA in 20 pA increments, 1 s duration). The trace in **(A)** shows a tonic firing response, **(B)** shows an initial response, **(C)** shows delayed spike response, **(D)** shows single spike response and in **(E)** is irregular spike response.

The firing pattern of action potential discharges evoked by intracellular current pulses in GABA neurons included five distinct groups: tonic, initial, delayed, single, and irregular (Prescott and De Koninck, 2002; Schoffnegger et al., 2006). Representative examples are shown in **Figure 2**. Tonic firing cells showed continuous, regular action potential discharges throughout the depolarization interval (**Figure 2A**). Initial cells showed a self-limiting burst of action potential discharges at the onset of depolarization (**Figure 2B**). Delayed firing cells had a prolonged non-spiking interval prior to the first action potential discharge followed by a continuous string of action potentials to the end of the depolarization phase (**Figure 2C**). Single spike cells showed just that only one action potential discharge at the onset of depolarization (**Figure 2D**). Finally, irregular firing cells did not fit any of the categories described above (**Figure 2E**). All cells showed a graded action potential response with current magnitude except the single firing cells. The incidence of firing patterns in sham and CCI mice were pooled in **Table 1**. At first blush, the incidence of spontaneous activity (37.9 vs. 25.0%) and the incidence of initial spike discharge pattern (20.7 vs. 7.1%) seemed higher in sham mice versus CCI mice; whereas the incidence of a delayed spike burst pattern (21.4 vs. 10.3%) single spike response (14.3 vs. 6.9%) and irregular spike response (21.4 vs. 13.8%) all seemed higher in CCI mice than in sham mice. Yet, none of these distributions achieved statistical significance with the samples sizes used.

### Physiological Properties of EGFP-GAD67- Neurons in CCI Mice

**FIGURE 3:**
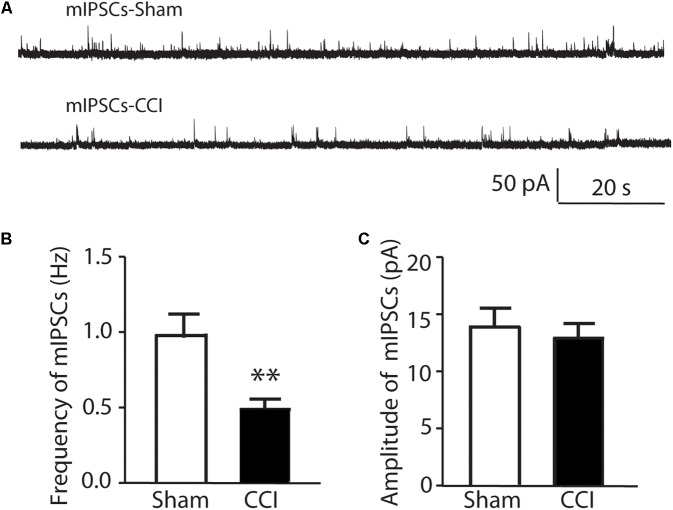
The comparison of miniature IPSCs in CCI and sham injury mice are shown. In **(A)** representative analog recordings are shown for mIPSCs in sham injury mice (mIPSCs-Sham, top) and CCI mice (mIPSCs-CCI). The bar graphs in **(B)** show that the mean frequency of mIPSCs was significantly reduced in CCI mice compared to sham control mice while the bar graphs in **(C)** show that the mean amplitude of mIPSCs was not different between CCI and sham mice. ^∗∗^*p* < 0.01.

A difference in the physiological properties of EGFP negative SG cells in CCI versus sham mice was observed in an analysis of miniature inhibitory post-synaptic currents (mIPSCs). The mIPSCs were recorded from 16 non-identified lamina II neurons in sham surgery mice and from 14 non-identified lamina II neurons in CCI mice 10 min following in the addition of 0.5 μM TTX to the recording bath. As shown in **Figure 3** the frequency of mIPSCs is 0.99 ± 0.15 Hz in sham mice and reduced to 0.48 ± 0.08 Hz in CCI mice (**Figures 3A,B**). The amplitude of mIPSCs showed no change between sham and CCI mice (**Figures 3A,C**). No clear differences were apparent in the kinetics of individual mIPSCs that would suggest a difference in GABA- versus glycine-mediated mIPSCs in either group consistent with previous work (Zhang et al., 2013). Nevertheless, these data suggest that the inhibitory tone was reduced in spinal dorsal horn following CCI, though increased sample could have revealed more subtle differences.

### Morphological Changes of EGFP-GAD67+ Neurons in CCI Mice

To compare the morphological changes of GABA neurons in CCI and sham mice, a total of 31 EGFP-expressing neurons were filled with 0.5% neurobiotin during whole cell recording in parasagittal slices. 15 were from CCI mice and 16 were from sham mice.

The cells were classified according to the scheme of Grudt and Perl (2002). Roughly half the GABA cells in both CCI and sham mice showed an islet cells profile with a dendritic tree that was elongated in the rostrocaudal direction but very limited in the dorsoventral direction. The dendritic tree was essentially confined to lamina II. Roughly one quarter of cells showed a vertical cell profile characterized by a pronounced ventral orientation of their dendritic tree extending down into lamina III in all cases and deeper still in some. The last quarter of cells showed a radial profile with a round or polygonal cell body located in lamina II inner in six out of eight cases and with multiple (5–10) primary dendrites that radiated in all directions.

**FIGURE 4:**
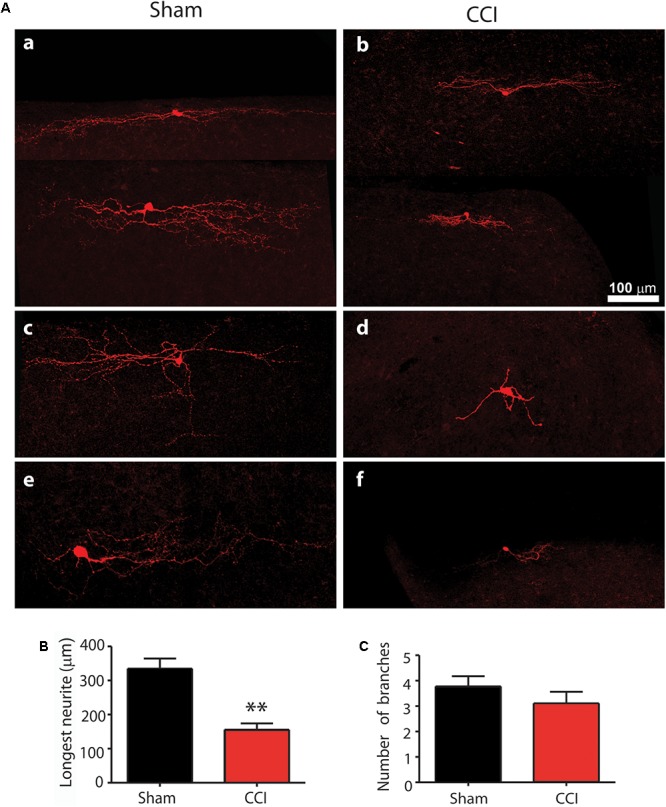
The comparison of morphological profile of GAD67+ neurons in CCI and sham injury control mice is shown. Representative confocal images of neurobiocytin filled neurons are shown in **(A)** for sham **(a,c,e)** and CCI mice **(b,d,f)**. The top line **(a,b)** show representative islet cells; the center line **(c,d)** shows representative vertical cells; and the bottom line **(e,f**) shows representative radial cells. The bar graph in **(B)** shows that the mean length of the longest neurite was significantly reduced in CCI mice (red bar) compared with sham mice (black bar). Finally, the bar graph in **(C)** shows that the mean number of branches from the longest neurite was not significantly different between CCI and sham mice.

There was no significant difference in the size of the soma of neurons between CCI and sham mice, however, there appeared to be clear differences in the extent of dendritic arbor in GABA cells of all profile types between the CCI and sham mice. To quantify these differences, the arbor length and the number of branches of EGFP-expressing neurons were analyzed. The arbor length and number of branches are variable between islet, vertical and radial cells and the most prevalent type was the islet cell group and so only these were quantified. A total of nine islet cells from sham and eight islet cells from CCI mice were analyzed. As shown in **Figures 4A,B**, the longest dendritic branch from sham mice averaged 334.94 ± 29.48 μm, while in CCI mice this was significantly less, averaging 160.92 ± 18.83 μm (*p* = 0.003). The total number of branches from the longest dendrite was not significantly different between the two groups of neurons (3.50 ± 0.31 verses 3.38 ± 0.4, **Figure 4C**). Representative examples of the reduced dendritic arbors in the vertical and radial cells are shown in **Figures 4Ac–f**.

## Discussion

Dysfunction of inhibitory interneurons with resulting increased excitability of spinal neurons has been proposed as a key mechanism underlying neuropathic pain (Sugimoto et al., 1990; Woolf and Mannion, 1999; Todd, 2015). However, the mechanisms underlying this remains controversial (Moore et al., 2002; Polgar et al., 2003). Pharmacological antagonists for either spinal GABA or glycine receptors results in behavioral signs consistent with neuropathic pain in otherwise naïve rats (Yaksh, 1989). A number of published studies concluded that a loss of GABA immunoreactivity in the dorsal horn (Castro-Lopes et al., 1993; Ibuki et al., 1997; Eaton et al., 1998) possibly due to a loss of GABA neurons occurs as a result of nerve injury as indicated by the presence of apoptotic markers in cells in the superficial dorsal horn (Moore et al., 2002; Scholz et al., 2005; Meisner et al., 2010). On the other hand, conflicting data indicated that neurons in laminae I to III were not lost in animals with either CCI (Polgar et al., 2003, 2004) or spared nerve injury (SNI) (Polgar et al., 2005). The apoptotic spinal cells that were seen in SNI were be microglia and not neurons (Polgar et al., 2005). Yet, pharmacological data shows that both GABA_A_ and GABA_B_ agonists reverse allodynia and hyperalgesia in models of neuropathic pain (Hwang and Yaksh, 1997; Malan et al., 2002), suggesting a deficit in GABA mediated inhibition following nerve injury.

Reduced excitatory drive to GABA neurons following loss of primary afferent input to these cells following nerve injury is an alternate explanation for the disinhibition that is observed (Leitner et al., 2013). GABA neurons in CCI rats showed reduced mEPSCs and increased paired pulse ratio to afferent stimulation; and this was also in the context of no change in the density and morphology of excitatory contacts upon GABA neuron dendrites (Leitner et al., 2013). It was concluded that presynaptic excitatory drive was reduced to GABA neurons following CCI injury. Yet, another study revealed no significant difference in the magnitude of EPSCs evoked by electrical stimulation of the dorsal root at C-fiber strength in CCI mice (Schoffnegger et al., 2006); and other (Moore et al., 2002) confirmed this in CCI as well as in SNI and partial nerve ligation where primary afferent-evoked EPSCs in lamina II neurons were not altered. Combined these results suggest there is no modification of excitatory input from primary afferents to GABA neurons in lamina II following nerve injury. Yet, the latter study just cited did reveal reduced primary afferent-evoked IPSCs in lamina II neurons (Moore et al., 2002). Similarly, the density of GAD65 inhibitory terminals in lamina I and lamina II were reduced after nerve injury (Lorenzo et al., 2014). In the current study, we found that the dendrites of GABA neurons significantly retract and this coincides with a decreased frequency of mIPSCs in spinal neurons in neuropathic mice. Given that excitatory inputs preferentially make contact to the distal dendrites while inhibitory inputs are concentrated nearer to the soma in lamina II neurons (Kato et al., 2007), retraction of distal dendrites could also result in reduced excitatory drive to GABA neurons without affecting the average density of the remaining excitatory synapses to these cells. It should be noted that other functions of the EGFP-GAD67- neurons following nerve injury was not tested. Combined, these findings suggest that part of the transition from acute to chronic pain is the physical re-alignment of neurons in the dorsal horn. It will be of considerable further interest to determine whether similar, or perhaps polar opposite, changes in morphology of cells of other functional types undergo change with nerve injury. An expansion of dendritic arbor of excitatory interneurons or projection neurons would be very intriguing.

## Author Contributions

All authors listed have made a substantial, direct and intellectual contribution to the work, and approved it for publication.

## Conflict of Interest Statement

The authors declare that the research was conducted in the absence of any commercial or financial relationships that could be construed as a potential conflict of interest.
